# Hemoadsorption in Children with Cytokine Storm Using the Jafron HA330 and HA380 Cartridges

**DOI:** 10.3390/jcm14186359

**Published:** 2025-09-09

**Authors:** Kamila Azenova, Vitaliy Sazonov

**Affiliations:** 1 Department of Medicine, School of Medicine, Nazarbayev University, Astana Z05K4F4, Kazakhstan; kamila.azenova@nu.edu.kz; 2 Department of Surgery, School of Medicine, Nazarbayev University, Astana Z05K4F4, Kazakhstan; 3 Pediatric Anesthesiology and Intensive Care Unit, National Research Center for Maternal and Child Health, University Medical Center, Astana Z05K4F4, Kazakhstan

**Keywords:** sepsis, pediatric sepsis, hemoperfusion, hemadsorption, HA330/380

## Abstract

**Background:** A cytokine storm can lead to organ dysfunction and death in critically ill children. Extracorporeal hemoperfusion aims to reduce hyperinflammation by filtering out mid-range cytokines (e.g., IL-6), but pediatric data remain limited. **Methods:** We conducted a narrative review with PRISMA-guided screening of PubMed, Scopus, and Google Scholar for pediatric reports of HA330/HA380 from January 2020 to June 2025. Due to heterogeneity in populations, circuits, and outcome timing, the results were synthesized descriptively. Three studies met the inclusion criteria: a prospective series of 12 patients with septic shock using HA330, a single case of a pediatric heart transplant with HA380 during cardiopulmonary bypass, and a retrospective comparative cohort study of Pediatric Intensive Care Unit (PICU) oncology patients on continuous renal replacement therapy (CRRT) comparing HA330 (n = 11) versus CytoSorb (n = 10). **Results:** Three studies involving 23 pediatric patients were analyzed. The median age was 8 years, and 56.5% of patients were male. Most patients underwent hemoadsorption with HA330 via continuous renal replacement therapy (CRRT) or continuous venovenous hemodiafiltration (CVVHDF). Post-treatment reductions were noted in interleukin-6 (IL-6) (mean −69.6%), C-reactive protein (CRP) (−59.0%), and procalcitonin (PCT) (−70.4%). Severity scores (Pediatric Logistic Organ Dysfunction-2 (PELOD-2), Pediatric Risk of Mortality-3 (PRISM-3), and Pediatric Sequential Organ Failure Assessment (pSOFA) improved significantly (*p* = 0.002). The mean PICU stay was 15.6 days. The survival rate was 87%, and no hemoadsorption-related adverse events were reported. **Conclusions:** HA330/380 hemoadsorption is a safe and potentially effective treatment for pediatric cytokine storms, reducing inflammation and improving clinical status. However, larger, standardized studies are needed to confirm these findings and guide clinical use.

## 1. Introduction

A cytokine storm, also known as cytokine release syndrome (CRS), is a hyperinflammatory state involving the excessive and unregulated release of pro-inflammatory cytokines [1]. This response can be triggered by infections, malignancies, autoimmune diseases, or therapeutic interventions. A normal cytokine response is essential for host defense; however, pathological overexpression can result in endothelial dysfunction, capillary leak, coagulopathy, and, ultimately, multi-organ failure [2,3]. In children, a cytokine storm is most often triggered by severe infections, such as sepsis. However, it can also occur in settings such as with a postoperative systemic inflammatory response or oncological complications [4,5]. Sepsis is a major global health challenge and a leading cause of morbidity and mortality in pediatric intensive care units. The neonates and children under five years old are particularly vulnerable; one in seven deaths in this age group is attributed to sepsis [6]. Pediatric sepsis differs significantly from adult sepsis in its pathophysiology, clinical presentation, and response to treatment due to age-related variations in immune and organ function [7]. The recently established Phoenix Sepsis Score (PSS) improves recognition of sepsis in children by focusing on organ dysfunction as a key diagnostic feature [8].

The pathogenesis of sepsis and cytokine storm involves the excessive release of cytokines, including tumor necrosis factor-α (TNF-α), interleukin-1 (IL-1), interleukin-6 (IL-6), and interferon-γ [9,10]. These mediators promote vascular leakage, disseminated intravascular coagulation, and immune dysregulation [11,12]. Although the early administration of antimicrobials and supportive measures is the cornerstone of management, these strategies often fail to control the inflammatory cascade, particularly in patients with delayed recognition, multidrug-resistant pathogens, or underlying immunocompromise [13].

To address uncontrolled inflammation in sepsis and cytokine storms, extracorporeal blood purification strategies have been developed. Among these, hemoadsorption has emerged as a promising adjunctive therapy. Hemoadsorption cartridges remove circulating cytokines, endotoxins, and other inflammatory mediators directly from the bloodstream. This mitigates the systemic effects of hyperinflammation [14,15]. HA330 and HA380 cartridges (Jafron Biomedical Co., Ltd., Zhuhai, China) are synthetic resin-based adsorbers operating within a 10–60 kDa molecular weight range. These cartridges have demonstrated efficacy in removing IL-6, TNF-α, and other mid-sized molecules [16,17].

Although studies in adults have demonstrated clinical improvement with HA330/380-based hemoperfusion, data on its use in children remain limited [18,19]. Due to the significant impact and unfavorable outcomes associated with pediatric sepsis and cytokine storm, it is crucial to evaluate the feasibility, safety, and therapeutic effectiveness of this intervention in children. Pediatric patients, particularly those weighing less than 10 kg, present unique challenges in terms of circuit volume, hemodynamic tolerance, and anticoagulation strategy. Thus, evaluating hemoadsorption devices in this population is critical [20,21].

Study Aim. Given the significant impact of pediatric sepsis and the increasing focus on extracorporeal blood purification, this review aims to summarize the available data on the use of HA330 and HA380 hemoperfusion devices for treating cytokine storm in children, especially in cases of sepsis and septic shock. Specifically, we evaluate the devices’ clinical effectiveness, impact on inflammatory markers, and safety profile. We also outline the current limitations of the evidence and key directions for future research.

## 2. Materials and Methods

This literature review assessed the clinical effectiveness and safety of hemoadsorption therapy using HA330 and HA380 cartridges (Jafron Biomedical, Zhuhai, China) in pediatric patients with cytokine storm, primarily in cases of sepsis or septic shock. While this is a narrative review, the process adhered to the general principles of the PRISMA (Preferred Reporting Items for Systematic Reviews and Meta-Analyses) framework to ensure transparency and reproducibility.

We searched the primary biomedical databases PubMed and Scopus because of their comprehensive coverage of peer-reviewed literature. Additionally, we screened Google Scholar as a supplementary source to identify potentially relevant gray literature, early online publications, and studies not yet indexed in PubMed or Scopus. Although Google Scholar is less structured and more time-consuming to search, it occasionally identifies unique records, justifying its inclusion. Keywords were selected to capture studies addressing pediatric populations specifically and the use of HA330 or HA380 devices. These included combinations such as “HA330 and children,” “HA380 and children,” “hemoadsorption and pediatric sepsis,” and “cytokine storm and HA330.” The search covered the period from January 2020 to June 30, 2025, and was limited to English-language articles. Additionally, the reference lists of all retrieved full-text articles were manually screened to identify other potentially relevant studies that were not captured in the initial search.

We included clinical studies of pediatric patients (<18 years) treated with HA330 or HA380 hemoadsorption for sepsis, septic shock, cytokine storm, or postoperative systemic inflammatory response. Eligible designs were prospective/retrospective cohorts, case series, and case reports with extractable patient-level or group-level outcomes. We excluded adult-only studies, non-human studies, non-HA330/HA380 devices without pediatric subgroup data, editorials/letters without original data, and conference abstracts without full data. When pediatric data were embedded in mixed-age reports, pediatric results needed to be separately extractable. If multiple publications reported overlapping cohorts, we used the most complete data source. In the included publications, the term hemoperfusion was occasionally used to describe treatment sessions; for consistency, we use hemoadsorption throughout this review.

**Figure 1:**
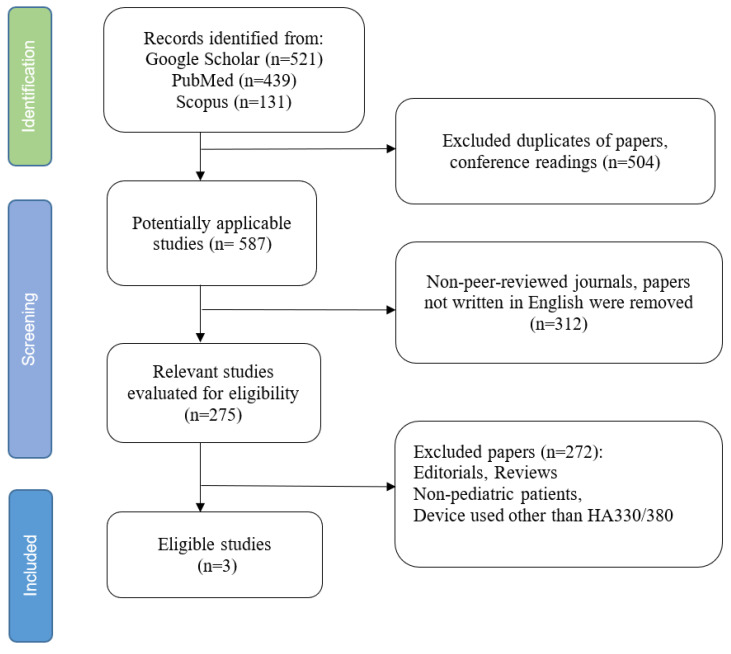
Flowchart of study selection.

All identified articles were initially screened by title and abstract. Full texts were then reviewed in detail to assess eligibility. Two reviewers performed this process independently, resolving disagreements through discussion and consensus. Due to significant heterogeneity in patient populations, extracorporeal configurations, and timing of outcomes, we planned a descriptive synthesis instead of quantitative pooling. Figure 1 presents a flow diagram summarizing the screening and selection process. Due to the narrative design and absence of quantitative synthesis, the review was not prospectively registered.

Risk of Bias Assessment. The quality of the included studies was evaluated using tools matched to the design of each study. Prospective and retrospective studies were appraised using the ROBINS-I and the Newcastle–Ottawa Scale (NOS). The case report was assessed using the JBI/CARE checklist. Two reviewers performed the assessments independently, resolving disagreements by consensus. The results of these assessments are summarized in Supplementary Table S2.

A pre-piloted extraction form was used by two reviewers to collect study design, setting, patient demographics (age, sex, and weight), indication, device (HA330/HA380), extracorporeal configuration (Continuous Renal Replacement Therapy (CRRT)/Continuous Venovenous Hemodiafiltration (CVVHDF)/Cardiopulmonary Bypass (CPB)/Extracorporeal Membrane Oxygenation (ECMO), anticoagulation strategy, priming solution/volume, session timing, frequency, duration, and outcomes. Authors were not contacted for missing data. When outcomes were reported only as medians (Interquartile Range (IQR), we extracted medians; means (SD) were extracted when available. The primary outcome was change in IL-6 from baseline to the closest post-treatment timepoint (preferentially 24–72 h). Secondary outcomes included Procalcitonin (PCT), CRP, clinical indices, pediatric severity scores (Pediatric Logistic Organ Dysfunction-2 (PELOD-2), Pediatric Risk of Mortality-3 (PRISM-3), and Pediatric Sequential Organ Failure Assessment (pSOFA), PICU length of stay (PICU-LOS), mechanical ventilation duration, survival, and device-related adverse events.

Due to the small number, clinical and methodological heterogeneity regarding population, extracorporeal circuits, session “dose” and timing, and outcome definitions, we prespecified a descriptive synthesis without quantitative pooling. Where two or more studies reported comparable time points and metrics, we presented the study-level summary statistics side by side. We did not plan any subgroup or meta-regression analyses a priori. According to the criteria, three papers with a total of 23 pediatric cases were collected in this review.

## 3. Results

After screening and selecting the studies, this review included three studies, yielding a total of 23 pediatric patients who received hemoadsorption therapy using HA330 or HA380 cartridges for sepsis, septic shock, or systemic inflammatory response syndrome (SIRS). The designs were as follows: one prospective series, one comparative cohort, and one case report. The devices included the HA330, which was used for intermittent sessions, and the HA380, which was used for continuous adsorption during cardiopulmonary bypass (CPB).

The patients’ ages ranged from 6 months to 15 years, with a median age of 8 years. The majority were male (n = 13; 56.5%).

Most patients had underlying comorbid conditions, with acute lymphoblastic leukemia (ALL) being the most frequently reported diagnosis (n = 6; 26.1%). The infectious etiologies of sepsis varied across studies. In one study, five patients (21.7%) developed sepsis in the context of a SARS-CoV-2 infection, while others had sepsis related to gastrointestinal or hepatobiliary infections (13.0%) or invasive pulmonary aspergillosis (8.7%). Single cases were attributed to *Staphylococcus epidermidis* and *Escherichia coli*. For several patients, the exact infectious source was not identified or reported. One patient developed systemic inflammatory response syndrome (SIRS) following cardiac surgery and underwent hemoadsorption using the HA380 cartridge integrated into the CPB circuit.

The remaining patients received hemoadsorption with the HA330 cartridge, which was incorporated into extracorporeal circuits, including CRRT in 52.2% of cases and CVVHDF in 43.5% of cases. Most of the extracorporeal circuits were primed with normal saline solution (NSS), which was used for 87.0% of patients. Most patients (n = 13; 56.5%) underwent two hemoadsorption sessions, while others received a single session.

The prespecified outcomes were IL-6, PCT, and CRP at fixed time points (preferably 72 h) as well as clinical indices. Reduction rates ranged from 43.5% to 71.2% for CRP, from 43.4% to 80.9% for procalcitonin PCT, and from 68.0% to 85.6% for IL-6. One study, which included 12 patients, only reported median values, which limited the ability to calculate pooled averages. For the remaining 11 patients across three studies, the mean reduction rates were calculated as follows: 69.6% for IL-6, 59.0% for CRP, and 70.4% for PCT.

In a pediatric oncology cohort on CRRT (HA330, n = 11; CytoSorb, n = 10), the percentage changes in CRP, IL-6, and PCT were not significantly different between devices (all *p* > 0.5). Survival and clinical indices were also comparable at the reported time points. However, interpretation requires caution due to non-equivalent exposure (intermittent 2–4 h HA330 sessions, often repeated the next day, versus ~24 h continuous CytoSorb sessions).

Two studies provided information on clinical severity scores. One study showed a nearly fivefold reduction in the PELOD-2 score post-treatment (*p* = 0.002), while the PRISM-3 score decreased from a median of 16.5 to 5.5 (*p* = 0.002), reflecting a significant reduction in mortality risk. Another study reported a decrease in pSOFA scores from a mean of 16.6 to 3.5 (*p* = 0.003), indicating significant improvement in overall organ function following hemoadsorption.

In the prospective series, the vasoactive inotropic score (VIS) and lactate improved from 17.5 (IQR: 9.8–46.0) at baseline to 1.5 (IQR: 0–6.0) at 72 h (*p* = 0.003), and from 3.1 (IQR: 1.9–5.1) to 1.7 (IQR: 1–2.3) at 72 h (*p* = 0.03), respectively.

The average PICU-LOS across studies was 15.6 days. Data on mechanical ventilation length of stay (MV-LOS) were limited, with only one study providing detailed values. Among 15 patients, the mean duration of mechanical ventilation was 9.5 days. Due to heterogeneity in populations and exposure dose, we do not pool LOS or survival across studies.

Overall, 20 out of 23 patients (87%) survived and were discharged from the PICU. Hemoadsorption was technically feasible with both CRRT and CPB configurations. There were no reported device-related adverse events. The three fatalities were attributed to progression of underlying conditions rather than to adverse effects of hemoadsorption. Table 1 summarizes the detailed clinical characteristics and outcomes of the included patients.

Risk of Bias. Overall, the included studies were of moderate to serious risk of bias, primarily due to small sample sizes, the absence of control groups, and the potential for confounding factors introduced by co-interventions. The single case report was rated as having a high risk of bias due to its anecdotal nature. Full assessments by domain are provided in Supplementary Table S2.

## 4. Discussion

The objective of this literature review was to evaluate the clinical effectiveness and safety of HA330/380 hemoadsorption cartridges when used as an adjunctive therapy for pediatric patients experiencing a cytokine storm, primarily due to sepsis or septic shock. Across the reviewed studies, most patients showed clinical improvement and a reduction in inflammatory markers. However, the generalizability of these results is limited by small sample sizes, heterogeneity in clinical protocols, and inconsistencies in outcome reporting. Nevertheless, several critical insights emerge.

### 4.1. Main Findings

One of the most consistent observations across studies was the significant reduction in inflammatory biomarkers following hemoadsorption. Reductions in IL-6, PCT, and CRP ranged from 68% to 86%, 43% to 89%, and 43% to 72%, respectively. Although CRP has a relatively high molecular weight (approximately 120 kDa) and is theoretically beyond the adsorptive range of HA330/380 cartridges (10–60 kDa), CRP fell likely due to upstream IL-6 suppression rather than direct adsorption [24,25].

In a retrospective cohort (Ryazanova et al. [23]), short-session HA330 produced similar reductions to those achieved with continuous CytoSorb. This finding has implications for reducing extracorporeal exposure time in critically ill children. However, non-equivalent exposure limits the ability to draw conclusions about the device. Several reports have described declines in creatinine, Aspartate Aminotransferase/ Alanine Aminotransferase (AST/ALT), and Gamma-Glutamyl Transferase (GGT) levels after treatment. However, these changes are exploratory and may reflect concurrent clinical recovery and co-interventions rather than a direct adsorption effect [5]. Thus, they should not be over-interpreted. It should be noted that these findings were not consistently reported.

Clinical severity scores, available in two of the three reviewed studies, demonstrated significant improvement after hemoadsorption. The PELOD-2 and PRISM-3 scores reported by Siripanadorn and Samransamruajkit [22] decreased markedly within 72 h post-intervention, indicating a transition from moderate-to-severe to mild organ dysfunction and a lower risk of mortality. Similarly, the pSOFA score in Ryazanova et al.’s [23] study declined from 16.6 to 3.5, supporting the clinical relevance of cytokine removal in improving organ function.

Notably, hemoadsorption was well tolerated in all studies. Of the 23 children treated, 20 survived and were discharged, and 3 deaths were attributed to underlying diseases rather than to the procedure itself. There were no device-related adverse events reported, and circuit-related issues were uncommon, which reinforces their biocompatibility and procedural safety.

Caution is required when interpreting results across devices [26]. In the comparative cohort, CytoSorb was typically used for 24 h of continuous contact, whereas HA330 was used for intermittent 2–4 h sessions, often repeated the next day. Therefore, apparent similarity in short-term biomarker reductions reflects non-equivalent exposure [27]. To enable fair cross-device comparisons and disentangle dose from device effects, future pediatric studies should adopt dose-equivalence reporting (e.g., contact hours × effective surface area).

A pediatric cytokine storm is characterized by the sustained activation of the innate immune system, resulting in high levels of circulating mid-range mediators (e.g., IL-6), which amplify endothelial injury, capillary leak, and vasoplegia [3]. In this setting, the theoretical appeal of hemoadsorption is not “pan-cytokine removal,” but rather the attenuation of the highest peaks over a limited time period. This allows conventional therapies, such as source control, antibiotics, vasopressors, and fluid stewardship, to regain effectiveness. This reframing helps explain why the most consistent signals appear within 24–72 h, and why benefits often align with VIS and lactate trajectories rather than with single-biomarker nadirs.

### 4.2. Interpretive Considerations and Clinical Nuances

Several factors may influence the interpretation of these outcomes. One important variable is the immunocompromised status of many patients, especially those undergoing treatment for hematologic malignancies [23,28]. Immunosuppressed children may respond differently to cytokine modulation, and more research is needed to determine whether hemoadsorption provides comparable benefits to individuals with different immune profiles.

The timing of hemoadsorption initiation is another critical factor. While the current expert consensus for adult patients is to initiate treatment within 24 h of diagnosis or biomarker elevation, pediatric data remain limited [29,30,31]. Many reports initiated hemoperfusion late, after conventional therapies had failed. However, given the rapid improvements in biomarkers and VIS observed within 24–72 h, earlier initiation is biologically plausible. Hypothesis-generating triggers could include rising IL-6 despite source control and antibiotics, VIS ≥ 15, or failure of lactate to clear after initial resuscitation. This practice may reduce the procedure’s effectiveness, underscoring the need to incorporate hemoadsorption earlier in the treatment algorithm as part of a multimodal sepsis management strategy rather than as salvage therapy.

Additionally, session frequency and duration varied across studies, adding to clinical heterogeneity. While many patients received one or two sessions, the rationale for repeating therapy was not consistently described. For instance, Sazonov et al. [28] administered a second session five days after the first due to clinical deterioration. This emphasizes the importance of continuous monitoring of biomarkers and clinical status to inform decisions about repeated hemoadsorption [32].

Hemoadsorption was feasible with CRRT (CVVHDF)-integrated circuits and during cardiopulmonary bypass. When CRRT membranes with intrinsic adsorptive properties (e.g., AN69-based) were used, an additional clearance effect was observed with HA330 compared to CRRT alone [16,33]. These configurations support the practical integration of HA cartridges into standard pediatric extracorporeal workflows, but they also highlight the importance of considering co-adsorption when attributing effects [23].

Furthermore, HA330/380 cartridges have demonstrated compatibility with various extracorporeal systems, including ECMO and CPB circuits [5,34,35]. This highlights their technical versatility. This flexibility allows for their integration into personalized, context-specific critical care strategies for pediatric patients.

Although declines in IL-6, PCT, and CRP are encouraging, a clinically meaningful benefit hinges on hemodynamics and organ support, including lower VIS, lactate clearance, fewer days on ventilation, and a shorter PICU stay. Future reports should align clinical assessments and sampling at 0, 24, 48, and 72 h. They should also predefine minimally important differences (e.g., VIS decrease of ≥5 within 24–48 h) and present denominators at each time point to avoid silent attrition bias.

### 4.3. Safety Considerations in Pediatrics

In addition to evaluating the efficacy of hemoadsorption in children, it must also be critically appraised for safety. This is because circuit-related risks and pharmacokinetic consequences are magnified in patients with low body weight.

For infants and children weighing close to 10 kg, the priming volume can approach a significant portion of the circulating volume, which can lead to hemodynamic instability [16]. Borankulova and Sazonov (2024) [20] noted that mortality in infants receiving hemoadsorption was nearly 50%, primarily due to circulatory compromise. Strategies include red blood cell (RBC)/albumin priming when extracorporeal volume exceeds ~10% of estimated blood volume, gradual flow ramp-up, and meticulous volume accounting. Dedicated low-volume cartridges could significantly increase the number of patients who are eligible for this procedure.

Some drugs (e.g., certain antibiotics) may be partially absorbed during hemoperfusion, particularly with prolonged contact or high flow rates [14,36]. Therapeutic drug monitoring and dose adjustment should be considered, particularly in small children with limited pharmacokinetic reserve, where clinically relevant.

Anticoagulation is another critical issue. Although heparin was used in most of the reviewed cases, it carries the risk of bleeding and heparin-induced thrombocytopenia (HIT) and other complications [37]. In contrast, citrate anticoagulation is a safer alternative because of its regional action and lower systemic toxicity [38,39]. However, citrate accumulation may lead to metabolic complications in children with liver dysfunction [40,41]. Despite these risks, several studies suggest that, with careful monitoring, citrate can be safely used even in patients with liver compromise [42]. It is advisable to use protocolized selection and monitoring aligned with circuit size, weight, and hepatic function [43].

During higher-exposure regimens, adsorptive removal of antibiotics, sedatives, analgesics, and select antifungals is plausible. To avoid under- or over-treatment, we recommend therapeutic drug monitoring where available, dose re-titration after sessions, and BIS/RASS-guided sedation. For low-weight children, pair these recommendations with meticulous fluid and calcium balance monitoring (if citrate is used) and explicit documentation of device-related adverse events versus general circuit issues.

### 4.4. Limitations and Future Perspectives

Despite the preliminary findings being promising, this review has several limitations that must be acknowledged. Most notably, the small number of studies and the limited patient population (n = 23 across three articles) substantially restrict the generalizability of the results. The small sample size precludes robust statistical analysis and increases the risk of bias. Larger, multicenter studies are needed to validate these early findings and provide definitive evidence of efficacy and safety.

A second limitation lies in the heterogeneity of study designs. Variability in patient age, weight, underlying conditions, timing of hemoadsorption initiation, the number of treatment sessions, and the types of extracorporeal circuits complicates the comparison of outcomes. Additionally, inconsistent reporting of data using standardized clinical scores or inflammatory markers makes it difficult to draw consistent conclusions across the cohort.

The lack of statistical analysis is another important limitation. Because many of the included studies were case reports or small series and some only provided median values without raw data, it was not possible to conduct pooled analyses or calculate effect sizes. Consequently, the strength of the evidence remains low, and the findings should be interpreted cautiously.

Another major limitation is the absence of randomized controlled trials (RCTs). While RCTs in pediatric critical care are challenging due to ethical, logistical, and clinical constraints, their absence makes determining causality and controlling for confounding factors difficult. The dynamic nature of sepsis and the need for rapid intervention further complicate trial design. Nevertheless, well-designed prospective cohort studies or pragmatic trials could provide valuable insights.

The evidence base is limited by the small size and non-randomized design of the studies, the heterogeneity of exposure “dose,” and the inconsistent reporting of timepoints. The *p*-values reported in very small studies should be interpreted cautiously. Effect sizes and standardized trajectories at fixed time points are more informative. Prospective multicenter studies should prespecify dose equivalence, co-intervention capture, and a pediatric core outcome set.

Moving forward, pediatric hemoperfusion research should focus on pragmatic trials that incorporate clear dose-equivalence metrics (e.g., contact hours × surface area) for early use. Currently, exposure differences complicate the interpretation of results across devices. Protocols must be weight-stratified with tailored priming and anticoagulation approaches for small children to minimize hemodynamic risk. Subgroup analyses are especially needed for oncology patients with neutropenia and pathogen-specific infections, and for those managed with CRRT versus non-CRRT circuits, since these groups may respond differently. Finally, a multicenter pediatric registry with a harmonized core outcome set is needed. This set should include fixed-time-point biomarker assessments (IL-6, PCT, and CRP), clinical indices (VIS and pSOFA), ventilator-free days, PICU length of stay, and predefined adverse events. Such a registry would transform the current anecdotal evidence into generalizable, guideline-ready data.

Adoption will depend not only on physiologic effects but also on team expertise, device availability, and cost-effectiveness relative to alternative adjuncts. Pragmatic studies should track resource utilization, such as the number of cartridges used, the amount of time nurses spend on the procedure, and the amount of blood products used for priming. These studies should also evaluate access equity, as infants and low-weight children face unique barriers related to extracorporeal volume and monitoring capacity.

## 5. Conclusions

Hemoadsorption with HA330/HA380 is a promising adjunctive therapy for pediatric cytokine storms, mainly in cases of sepsis. It has been shown to substantially reduce IL-6, PCT, and CRP levels, while also improving clinical outcomes. There have been no reports of serious adverse events related to the device. The cartridges can be integrated with CRRT/CVVHDF, ECMO, and CPB; however, priming volume remains a concern in infants and children with low body weight (thus supporting the exploration of smaller cartridges, such as HA60).

However, evidence remains limited due to small, heterogeneous cohorts, non-standardized protocols, the absence of randomized trials, and scarce long-term data. Therefore, definitive conclusions regarding efficacy and safety are premature. Priorities include multicenter studies with standardized exposure and reporting (e.g., dose equivalence, fixed time points, and core outcomes), and work to refine timing, duration, and patient selection. Additionally, there is a need for pediatric-specific devices and anticoagulation strategies.

Overall, hemoperfusion should complement, not replace, standard care, and its use should be embedded within protocolized pediatric critical care pathways while higher-quality evidence is generated.

## Figures and Tables

**Table 1:** Summary of clinical parameters and hemadsorption therapy outcomes of patients across reviewed studies.

Authors	Siripanadorn and Samransamruajkit (2023) [22]	Napoleone et al. (2024) [5]	Ryazanova et al. (2024) [23]
Study design	Prospective observational	Single pediatric CPB case report	Retrospective, oncology cohort
Number of patients, n	12	1	10
Age, years (Median)	9.5	10	6.5
Sex (M/F)	M: 8; F: 4	M	M: 4; F: 6
Comorbidities /Inflammatory response caused by (underlying disease)	COVID-19; GI and hepatobiliary infection; Invasive pulmonary aspergillosis	Single ventricle/SIRS caused by surgical intervention	ALL; Sarcoma; AML; Pure red cell aplasia
Severity score before the procedure (If reported)	PELOD-2: 9.67 ± 3.90; Median = 9.5 PRISM-3: 18.08 ± 3.90; Median = 16.5	Not reported	pSOFA: 16.6 ± 3.16
Hemoadsorption therapy initiation time after diagnosing sepsis	Within 24 h	Not reported	Not reported
Device/Circuit	HA330/CRRT, ECMO in 1 patient	HA380/CPB	HA330/CVVHDF
Priming	NSS; 5% albumin in two cases	Fresh frozen plasma (FFP) 200 mL, albumin 20% 50 mL, and ringer acetate 800 mL	NSS; 2 patients were replaced to red blood cell suspension
Inflammation markers levels before hemoadsorption	Median (Interquartile Range (IQR) IL-6: 206.4 (111–412.5) pg/mL; CRP: 87.6 (16.3–135.2) mg/L; PCT: 8.4 (1.3–21.6) pg/mL	IL-6: 146 pg/mL; CRP: 169 mg/L; PCT: 10.88 ng/mL	(mean ± SD) IL-6: 539.89 ± 475.02 pg/mL; CRP: 288.17 ± 99.34 mg/L; PCT: 245.10 ± 115.73 ng/mL
Number of sessions, n/Hemoadsorption duration time	24 sessions: 2 sessions per patient/2–4 h per patient	1 session/218 min (3 h 38 min)	11 sessions; 1 session per patient; one pt had 2 sessions/4 h per patient
Inflammation marker levels after hemoadsorption	(median (IQR) IL-6: 37.4 (6.4–146.8) pg/mL; CRP: 25.2 (6.8–120.4) mg/L; PCT: 1.6 (1.1–8.8) pg/mL	IL-6: 21 pg/mL; CRP: 95.5 mg/L; PCT: 6.16 ng/mL	(mean ± SD) IL-6: 107.7 ± 52.21 pg/mL; CRP: 105.63 ± 49.32 mg/L; PCT: 11.89 ± 13.18 ng/mL
Reduction rate, %	IL-6: −81.89; CRP: −71.23; PCT: −80.95	IL-6: −85.62; CRP: −43.49; PCT: −43.38	IL-6: −68.02 ± 19.78; CRP: −60.596 ± 19.51; PCT: −73.06 ± 23.79
Severity score after the procedure (If applicable)	PELOD-2: ~2 (median) PRISM-3: ~5.5 (median)	Not reported	pSOFA: 3.5 ± 1.17
Hospital stay metrics (PICU-LOS/MV-LOS), days (mean ± SD)	PICU-LOS: 15.58 ± 11.08 MV-LOS: 8.75 ± 9.37	PICU-LOS: 15 MV-LOS: N/A	PICU-LOS: 15.7 ± 4.69 MV-LOS: 2–3
Outcome (Discharged/Fatal)	Discharged: 10; Fatal: 2	Discharged	Discharged: 9; Fatal: 1

Note. ALL—acute lymphoblastic leukemia; SIRS—systemic inflammatory response syndrome; AML—acute myeloid leukemia; PELOD-2—Pediatric Logistic Organ Dysfunction-2; PRISM-3—Pediatric Risk of Mortality-3; pSOFA—Pediatric Sequential Organ Failure Assessment; CVVHDF—continuous venovenous hemodiafiltration; NSS—normal saline solution; CRRT—continuous renal replacement therapy; CPB—cardiopulmonary bypass; FFP—fresh frozen plasma; IL-6—interleukin-6; CRP—C-reactive protein; PCT—procalcitonin; PICU-LOS—pediatric intensive care unit–length of stay; MV-LOS—mechanical ventilation–length of stay.

## Data Availability

No new data were created or analyzed in this study. Data sharing is not applicable to this article.

## Supplementary Materials

The following supporting information can be downloaded at: https://www.mdpi.com/article/10.3390/jcm14186359/s1, Table S1: Glossary of Abbreviations; Table S2: Risk of bias.

## Abbreviations

The following abbreviations are used in this manuscript:

ALL	Acute Lymphoblastic Leukemia
ALT	Alanine Aminotransferase
AML	Acute Myeloid Leukemia
AST	Aspartate Aminotransferase
BIS	Bispectral Index
CARE	CAse REport (reporting checklist)
CPB	Cardiopulmonary Bypass
COVID-19	Coronavirus Disease 2019
CRP	C-Reactive Protein
CRRT	Continuous Renal Replacement Therapy
CRS	Cytokine Release Syndrome
CVVHDF	Continuous Venovenous Hemodiafiltration
ECMO	Extracorporeal Membrane Oxygenation
FFP	Fresh Frozen Plasma
GI	Gastrointestinal
GGT	Gamma-Glutamyl Transferase
HIT	Heparin-Induced Thrombocytopenia
IL-1	Interleukin-1
IL-6	Interleukin-6
IQR	Interquartile Range
JBI	Joanna Briggs Institute
LOS	Length of Stay
MV-LOS	Mechanical Ventilation–Length of Stay
NOS	Newcastle–Ottawa Scale
NSS	Normal Saline Solution
PCT	Procalcitonin
PELOD-2	Pediatric Logistic Organ Dysfunction-2
PICU	Pediatric Intensive Care Unit
PICU-LOS	Pediatric Intensive Care Unit–Length of Stay
PRISMA	Preferred Reporting Items for Systematic Reviews and Meta-Analyses
PRISM-3	Pediatric Risk of Mortality-3
pSOFA	Pediatric Sequential Organ Failure Assessment
PSS	Phoenix Sepsis Score
RBC	Red Blood Cell
RASS	Richmond Agitation–Sedation Scale
RCTs	Randomized Controlled Trials
ROBINS-I	Risk Of Bias In Non-Randomized Studies of Interventions
SARS-CoV-2	Severe Acute Respiratory Syndrome Coronavirus 2
SD	Standard Deviation
SIRS	Systemic Inflammatory Response Syndrome
TNF-α	Tumor Necrosis Factor-alpha
TDM	Therapeutic Drug Monitoring
VIS	Vasoactive Inotropic Score
