# Supplementary material for: Hemoadsorption in Children with Cytokine Storm Using the Jafron HA330 and HA380 Cartridges
Source: J Clin Med. 2025 Sep 9;14(18):6359. doi: 10.3390/jcm14186359 (PMC12470262; doi:10.3390/jcm14186359)
Supplement: Supplementary file 1 [file jcm-14-06359-s001.zip › jcm-3816011-supplementary.pdf]

Table S1. Glossary of Abbreviations

Abbreviation	Full term	Context in manuscript
**ALL**	Acute Lymphoblastic Leukemia	Patient populations and comorbid conditions reported in pediatric oncology cohorts
**ALT**	Alanine Aminotransferase	Laboratory parameters in safety and organ function monitoring
**AML**	Acute Myeloid Leukemia	Patient populations and comorbid conditions in pediatric oncology settings
**AST**	Aspartate Aminotransferase	Laboratory monitoring of hepatic involvement
**CPB**	Cardiopulmonary Bypass	Case report of hemoadsorption with HA380 during heart transplantation
**CRP**	C-Reactive Protein	Inflammatory biomarker outcomes across studies
**CRRT**	Continuous Renal Replacement Therapy	Extracorporeal circuit modality used for hemoadsorption in pediatric sepsis
**CVVHDF**	Continuous Venovenous Hemodiafiltration	Specific CRRT modality used in included studies
**CytoSorb**		Trade name of a porous polymer bead hemoadsorption cartridge (adult experience; limited pediatric data)
**ECMO**	Extracorporeal Membrane Oxygenation	Context of extracorporeal support in critically ill children
**FFP**	Fresh Frozen Plasma	Priming or transfusion products in small-volume circuits
**GGT**	Gamma-Glutamyl Transferase	Laboratory biomarker mentioned in liver function monitoring
**HIT**	Heparin-Induced Thrombocytopenia	Anticoagulation risks discussed in safety considerations
**IL-1**	Interleukin-1	Proinflammatory cytokine mentioned in background rationale
**IL-6**	Interleukin-6	Primary biomarker outcome across included studies
**IQR**	Interquartile Range	Statistical reporting of nonparametric outcome data
**LOS**	Length of Stay	General reporting of hospital course
**MV-LOS**	Mechanical Ventilation – Length of Stay	Duration of respiratory support outcomes
**NSS**	Normal Saline Solution	Priming fluid in extracorporeal circuits
**PCT**	Procalcitonin	Biomarker outcome used to assess inflammatory response
**PELOD-2**	Pediatric Logistic Organ Dysfunction-2	Severity scoring system applied in patient evaluation
**PICU**	Pediatric Intensive Care Unit	Clinical setting of included patients

Abbreviation	Full term	Context in manuscript
**PICU-LOS**	Pediatric Intensive Care Unit – Length of Stay	Outcome measure of intensive care stay
**PRISM-3**	Pediatric Risk of Mortality-3	Severity scoring system for risk stratification
**pSOFA**	Pediatric Sequential Organ Failure Assessment	Severity score tracked during treatment response
**PSS**	Phoenix Sepsis Score	Tool for sepsis severity assessment
**RBC**	Red Blood Cell	Used in priming solutions for pediatric circuits
**RCTs**	Randomized Controlled Trials	Referenced in discussion of broader evidence
**SARS-CoV-2**	Severe Acute Respiratory Syndrome Coronavirus 2	Context of cytokine storm pathophysiology
**SD**	Standard Deviation	Statistical reporting of normally distributed data
**SIRS**	Systemic Inflammatory Response Syndrome	Clinical context of hyperinflammation
**TNF-α**	Tumor Necrosis Factor-alpha	Proinflammatory mediator discussed in background
**TDM**	Therapeutic Drug Monitoring	Recommended safety practice due to possible drug adsorption
**VIS**	Vasoactive Inotropic Score	Clinical index of cardiovascular support requirements

Table S2. Risk of bias

Study	Design	Tool used	Main limitations	Overall risk
Prospective cohort (12 pts, septic shock, HA330)	Prospective observational	NOS / ROBINS-I	No control group; small sample; multiple co-interventions	Moderate–serious
Retrospective oncology cohort (10 pts, HA330 vs CytoSorb)	Retrospective comparative	NOS / ROBINS-I	Non-randomized; small sample; dose/exposure not equivalent	Moderate–serious
CPB heart transplant case (HA380)	Case report	JBI / CARE checklist	Single patient; no comparator; anecdotal	High
